# Wear Rate, Tribo-Corrosion, and Plastic Deformation Values of Co-Cr-Mo Alloy in Ringer Lactate Solution

**DOI:** 10.3390/ma17102327

**Published:** 2024-05-14

**Authors:** Raimundo Nonato Alves Silva, Rui Neto, Angela Vieira, Priscila Leite, Polyana Radi, Carolina Hahn da Silveira, M. D. Santos, Filomena Viana, Lúcia Vieira

**Affiliations:** 1 Department of Materials Engineering, University of the State of Amazonas—UEA, Darcy Vargas, Manaus 69050-020, Brazil; rnasilva@uea.edu.br; 2 Department of Metallurgical and Materials Engineering, Faculty of Engineering, University of Porto—FEUP, Rua Dr. Roberto Frias, 4200-465 Porto, Portugal; fviana@fe.up.pt; 3 LAETA/INEGI—Institute of Science and Innovation in Mechanical and Industrial Engineering, Rua Dr. Roberto Frias, 4200-465 Porto, Portugal; 4 Department of Mechanical Engineering, Faculty of Engineering, University of Porto—FEUP, Rua Dr. Roberto Frias, 4200-465 Porto, Portugal; 5 Research and Development Institute (IP&D), University of Paraiba Valley (Univap), São José dos Campos 12244-000, Brazil; 6 Department of Mechanical Engineering, University of the State of Amazonas—UEA, Darcy Vargas, Manaus 69050-020, Brazil

**Keywords:** cobalt-based alloys, wear, tribocorrosion, orthopaedics, electrochemical

## Abstract

This study investigates the tribocorrosion performance of a cast Co-Cr-Mo alloy prepared using casting and electromagnetic stirring (EMS) at specific frequencies. The tribocorrosion behaviour of the alloy was evaluated when exposed to Ringer’s lactate solution to optimize the EMS parameters and improve its properties. The research focuses on biomedical implant applications and explores how EMS affects alloy wear and corrosion resistance. As did the friction coefficient and wear volume, the wear rate of samples produced with EMS frequencies of 75 Hz and 150 Hz decreased. These improvements are attributed to the ability of EMS to refine grain size and homogenize the microstructure, thereby increasing the resistance to tribocorrosion. Techniques such as scanning electron microscopy (SEM) and profilometry were used for surface and wear analysis, while mechanical properties were evaluated through instrumented indentation tests. The findings confirm that EMS improves the alloy’s durability and tribocorrosion resistance, making it highly suitable for demanding biomedical applications such as joint replacements. This highlights the importance of advanced manufacturing techniques in optimizing biomedical alloys for simulated body conditions.

## 1. Introduction

In orthopaedics, the behaviour of the union of tribocorrosion prostheses is exploited by many researchers to improve the performance of these devices, provide better quality of life to patients and avoid review surgeries [1]. 

Cobalt–chromium-based alloys have been developed and employed in highly demanding medical applications [2], such as in the knee and hip, due to their excellent ability to maintain mechanical properties and biocompatibility over long periods [3].

Co-Cr-Mo alloy castings or those forged with 28% Cr and 6% Mo could be divided into two orders according to the carbon content: (i) low-carbon alloys (C content < 0.05%) present a homogeneous structure and (ii) high-carbon alloys (the content of C > 0.15%) are characterized by carbide precipitation [4,5,6]. The function is to increase mechanical strength and, to a lesser extent, hardness [5]. The previous article used four parts for a numerical simulation, validated and produced per investment casting and characterization [3]. The first casting was produced without applying EMS for comparison. The others were submitted to different EMS frequencies for 15 min. In these three foundries, the applied EMS frequencies were 15 Hz, 75 Hz, and 150 Hz. 

On the other hand, the cast Co-Cr alloy has a coarse dendritic structure characteristic of investing casting and is strengthened by carbides, typically cast with ASTM F75 [7]. It combines mechanical properties and corrosion resistance [8,9,10]. Metallic alloy properties are determined by their microstructure, which can be enhanced by coating the surface, heat treatment, deforming the alloy, and controlling the processing conditions [11], such as electromagnetic stirring (EMS) or using solid solution. 

Namus et al. reported that the formation of ultrafine grains and nanocrystalline structures is not beneficial for these implants despite the good tribocorrosion behaviour of the alloy [10]. When comparing the corrosion rate results, the cast Co-Cr-Mo alloy showed much better values than the nanocrystalline alloy. Co-Cr-Mo alloy produced by process casting is sometimes subjected to a solution annealing procedure and forging, obtaining different microstructures with more hardness, strength, and wear resistance [8]. Thus, changes in the microstructure can affect the mechanical properties. However, microstructure and alloy composition affect corrosion behaviour in simulated body fluids due to changes in surface chemistry [12,13,14,15,16]. 

It is possible to produce Co-Cr-Mo alloys by various techniques, such as forging, powder metallurgy, SLM—selective laser melting—and investing casting [17]. The investing casting process is a well-known technology for manufacturing products with complex geometries, such as orthopaedic prostheses. However, there is a need for improvements in producing parts with refined grains and homogeneous microstructures. Electromagnetic stirring (EMS) is an effective method to obtain grain refinement and a homogenous refining structure [18,19]. It appears to be an excellent method for the manufacture of high-performance parts.

The friction and wear problems in the prosthesis for replacing hip joints and knees are mainly small-displacement wear (fretting wear) caused by metal-on-metal (MoM) contact, causing issues for prostheses users [5,20,21,22], and affecting the longevity of orthopaedic implants is the formation of wear debris, which has been linked to problems such as tissue inflammation, bone loss, and implant loosening [12]. The corrosion and the metallic ion release from implants made of these alloys are critical concerns in MoM articulations [2,23]. The wear reduces the life of components from the prosthesis, so the replacement cost increases [24].

In orthopaedics, the behaviour of a tribocorrosion regime joining prosthesis has been explored by many researchers, aiming to improve the performance of these devices, provide a better quality of life to patients, and avoid expensive review surgeries. Studies have evidenced the formation of a tribolayer on the surface of all kinds of metal joints [5,25,26]. This dynamic layer is a mixture of metallic oxides, metallic nanocrystals, and organic matter from joint fluids and functions as a solid lubricant. This tribolayer can be protective by reducing friction and wear on the metal surface [27]. According to Wimmer et al. [28], over 80% of the retrieved implants displayed a tribochemical layer on their surfaces, particularly on acetabular cups and femoral heads. Lyvers et al. [29] evidenced that molybdenum ions (Mo+) assist in the stability of the tribolayer under the tribocorrosion processes and show no cytotoxicity in vitro. 

Researchers use tribocorrosion to identify future problems in these materials. In other words, triboelectrochemical experiments or the degradation phenomena of material surfaces (wear, cracks, and corrosion) are subjected to mechanical loads (friction, abrasion, and erosion) and corrosion. This attack is caused by the environment (chemical and/or electrochemical interaction) by the simultaneous action of wear and corrosion [25,30,31]. When in contact with the body fluids, the layer can be removed by corrosion or tribocorrosion, which can release material particles [32,33]. In some cases, it is through the combined effect of these two abrasions and tribochemical degradation mechanisms. Reactions on articulated surfaces aim to modify in vivo wear behaviour [5]. 

In this context, several authors [8,34,35,36,37,38] have used saline solution, bovine serum, bovine calf serum (BCS), bovine serum albumin (BSA), distilled water, Hanks solution, and Phosphatase bovine serum (PBS)-like lubricating liquids in the simulation of corporate liquids. In the review paper, Shahini et al. [39] noted disagreements after using the electrolytes used in the experiments. These electrolyte PBS and BSA, in the tribocorrosion test, showed a lower coefficient of friction and corrosion products compared with having only PBS in the solution [40,41]. Albumin induces the generation of a tribofilm [36,42]. A lower corrosion rate was reported for the Co-Cr-Mo under a sliding test in the BCS solution compared with PBS due to protein existing in the BCS [43]. Because of the existence of chloride ions in the artificial saliva, the probability of more intense tribocorrosion is higher when compared with Ringer’s solution [44]. Herranz et al. [45] used three solutions (saline solution, PBS, and Ringer’s solution) to evaluate the behaviour of the Co-Cr Mo alloy when submitted to the tribocorrosion tests. They observed that Ringer’s solution was more corrosive than saline and PBS due to the high concentration of Cl^−1^. Metallic alloys, when implanted, suffer attacks from saline electrolytes that are highly oxygenated at a pH of about 7.4 and temperature near 37 °C, which can cause corrosion in metal; the human body is aggressive and can generate biomaterials to corrosion [46].

This study explores the behaviour of a Co-Cr-Mo alloy cast using investment casting with electromagnetic stirring using specific frequencies for the first time. It focuses on understanding how this cast alloy performs under tribocorrosion when exposed to a simulated biological fluid, precisely Ringer’s lactate solution. This innovative approach aims to enhance the alloy’s properties by optimizing the EMS parameters during the casting process.

## 2. Material and Methods

### 2.1. Co-Cr-Mo Alloy

The metal used in this study was medical-grade Co-Cr-Mo alloy, supplied by Zollern & Comandita, Maia, Portugal. The chemical composition of the Alloy was analysed at the Laboratory of INEGI—Institute of Science and Innovation in Mechanical Engineering and Industrial Engineering using the spectrometer Spectromaxx (AMETEK, Weiterstadt, Germany), shown in Table 1.

The alloy was melted in a vacuum furnace (INEGI, Porto, Portugal) equipped with high-frequency (50 kHz) induction heating in the melting chamber and electromagnetic stirring (EMS) in the pouring chamber [3]. The pouring of the Co-Cr alloy occurred at t = 373 s. Different electromagnetic frequency fields (EMS) were applied during the solidification of the Co-Cr-Mo alloy. The samples were separated into four samples: A1 (without frequency), A2 (15 Hz), A3 (75 Hz) and A4 (150 Hz). All Co-Cr-Mo samples were provided on a plate with 20.0 × 20.0 × 2 mm dimensions. After casting, the samples underwent no surface treatment—the material used in cast conditions. The previous work [3] discussed the process of obtaining the alloy which is used in the manufacture of orthopaedic prostheses.

The specimens were wet-ground using 280–2500 grit SiC paper. They were polished with diamond paste until they reached a mirror-like surface appearance (Ra ≤ 0.03 µm) using the standard metallographic procedure and then cleaned. Then, they were cleaned with acetone and distilled water for 5 min before the experiment.

### 2.2. Microstructural Analysis of Alloy Samples 

Four samples underwent microstructural analysis: A1 (without EMS application) and A2, A3, and A4, treated with electromagnetic stirring (EMS) frequencies of 15 Hz, 75 Hz, and 150 Hz, respectively. Post-tribocorrosion, the samples were meticulously cleaned using ultrasonic baths in propanol for 8 min, followed by distilled water for another 5 min. Preparation for analysis adhered to the Buhler protocol for cobalt-based alloys’ metallography. For characterizing the wear-affected surfaces, an optical microscope (DM 4000M, Leica Microsystems, Wetzlar, Germany) was employed, alongside scanning electron microscopy (Quanta 400 FEG ESEM, FEI, Dreieich, Germany) with an Energy Dispersive Spectroscopy (EDS) detector for detailed chemical composition analysis at localized areas.

**Figure 1:**
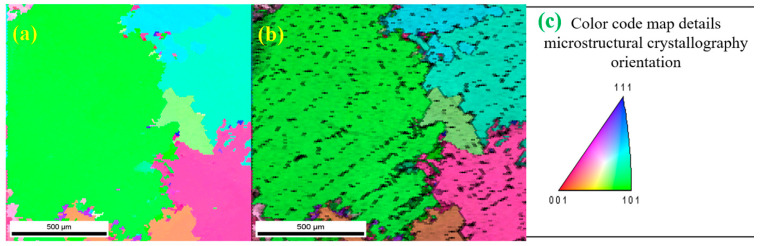
(**a**) Inverse pole figure (IPF) map shows alpha-Co; (**b**) inverse pole figure (IPF) + image quality (IQ); and (**c**) colour code map with microstructural crystallography.

Prior experiments had explored additional EMS frequencies (50 Hz, 100 Hz, and 200 Hz); however, the microstructural features observed remained consistent with those reported herein. Figure 1a displays the inverse pole figure (IPF) map, highlighting the alpha-Co grains’ varied crystallographic orientations and substantiating the presence of large grains. In Figure 1b, an overlaid image quality (IQ) map reveals carbide particles (depicted as black dots) distributed across the grains and grain boundaries (GB), enabling the clear delineation of dendritic structures. This detailed microstructural examination underscores the significant impact of EMS on grain orientation and size, contributing to our understanding of how varying frequencies can influence the alloy’s properties.

### 2.3. Mechanical Properties

Microhardness measurements were conducted to ascertain the mechanical properties of the alloy, using a force of 490.3 mN and a holding time of 15 s. The examination included assessments of hardness (H), the elastic modulus (E), and the ratio between hardness and the elastic modulus (H/E) for each sample (A1, A2, A3, and A4). These properties were gauged using the instrumented indentation technique, utilizing a Hysitron TI 950 Triboindenter machine (Bruker, Billerica, MA, USA). The process involved the use of a Berkovich tip under low load conditions (up to 10 mN), with calibration curves established prior to testing as per the manufacturer’s guidelines. This involved incrementally loading a fused quartz standard to define a calibrated response for penetrations ranging from 30 to 180 nm. During the indentation tests, periods of 5 s for loading, 5 s for dwell, and 2 s for unloading were adopted. A minimum of 16 indentations were performed across various regions of the samples, employing a load spectrum of 0.2 to 10 mN to ensure comprehensive data collection on penetration depth. Following this, the collected data and the corresponding load–displacement curves were meticulously analysed to derive the material’s elastic modulus (E) and hardness (H), offering insights into the mechanical integrity of the alloy under study.

### 2.4. Tribocorrosion Test

The tribological tests carried out in this experiment were performed in a reciprocating sliding tribometer, where wear tests obeyed a reciprocal linear slip on the samples to characterize friction behaviour with a sphere-on-plate contact geometry. The tribocorrosion test was performed in static and dynamic modes, using an electrochemical cell to measure the open circuit potential (OCP). The tests were performed at room temperature using a tribometer Ultra Micro UMT 2, Bruker, Coventry, UK, sphere-on-flat, following the ASTM G119 protocol [47]. The experiments were conducted and recorded, with average friction coefficient values and normal load according to the tribometer software. The effects of friction and corrosion were recorded simultaneously, as recommended by the tribocorrosion test [48] for sliding contacts. The electrochemical cell comprised a potentiostat, an Ag/AgCl reference electrode, and a platinum counter electrode. The working electrode was the sample analysed (A1, A2, A3, and A4), while the container was filled with 150 mL of Ringer lactate solution, was used to simulate normal physiological conditions, commercial lactated Ringer’s solution was used, acquired from Fresenius Kabi Brasil Ltd., this solution represented the level of salinity found in the human body, shown in Table 2, at an ambient temperature of 25 ± 2 °C and pH 6.0–7.5.

The tribological pairs were the surfaces of the samples (A1, A2, A3, and A4) positioned against an Al_2_O_3_ sphere (purchased from Ceraltec Cerâmica Técnica Ltd., Ibaté, SP, Brazil). The experimental parameters of the tribocorrosion tests are shown in Table 3. This time was selected in previous studies [33] to stabilize the OCP before the tests. 

A sphere of Al_2_O_3_, with a diameter of 4.76 mm and 99% purity, was used as a counterface to change the severity of the test. All tests were repeated twice and showed high reproducibility. The sphere was exchanged for each test. The total wear volume (plate) was measured using the wear profile of the optical profilometer (Taylor-Hobson Ltd., Leicester, UK). The chemical and topographic characterization of the samples and the wear tracks generated by the tribology tests were carried out via Scanning Electron Microscopy—SEM—the microstructure observation using a dispersive energy spectroscopy (EDS) system. Secondary and backscattered electrons were accelerated at 10 keV to evaluate the microstructural characteristics.

**Figure 2:**
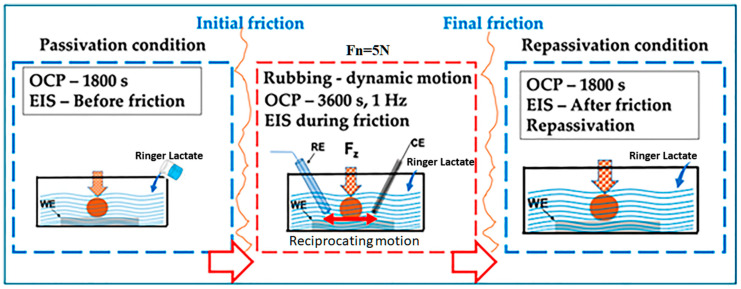
Schematic drawing of tribocorrosion cell showing the reference electrode (RE) Ag/AgCl, the platinum counter electrode (CE) and the working electrode (WE).

The OCPs test was run out for 1800 s before the friction test to allow for stabilization, during the friction test for 3600 s, and an additional 1800 s after the friction test to observe their stabilization behavior. The frictional force was measured using a load transducer. Qualitative and quantitative studies focusing on the interaction of the two processes, mechanical tribology, and electrochemical oxidation, have shown that the material degradation rate can be significantly or not-so-much affected. Figure 2 demonstrates the schematic drawing from the tribocorrosion cell, showing the three-step conditions: passivation, dynamic motion in a reciprocating way with OCP, and final passivation. During dynamic motion, a three-electrode electrochemical cell was used with the wear tester, the Ag/AgCl reference electrode (RE), a platinum counter electrode (CE), and the working electrode (WE). The results were analysed according to ASTM G3-14 [49].

The volumes of material removed on the wear track were calculated from these analyses. All tests were repeated at least two times to have repeatability regarding COF. The abrasive wear factor, K (mm^3^ N^−1^ m^−1^), was calculated using Equation (1):

Wear rate = Wv/Applied force × sliding distance(1)

where the wear rate is in mm^3^ N^−1^ m^−1^, Wv is the volume loss of the sample in mm^3^, the applied force load is in N, and the total sliding distance is in mm.

## 3. Results and Discussion

### 3.1. Microstructure

**Figure 3:**
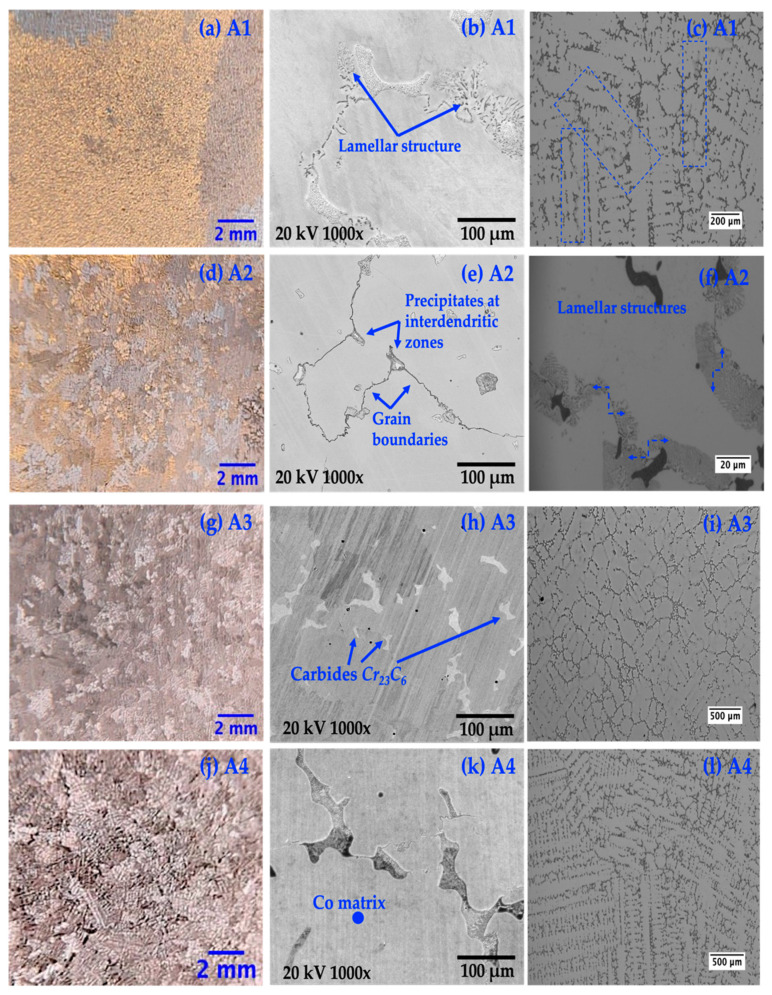
O.M images of Co-C-Mo alloy samples after foundry and SEM images. (**a**–**c**) A1 No EMS, (**d**–**f**) A2-15 Hz sample, (**g**–**i**) A3-75 Hz sample and (**j**–**l**) A4-150 Hz sample.

The ASTM F75 alloy under study exhibited a microstructure characterized by carbide colonies with a lamellar layout and a notably coarse grain structure, with sizes surpassing 1000 µm. This structure is apparent within the grains themselves and at their boundaries. Figure 3 highlights the macrostructures of samples treated and untreated with electromagnetic stirring (EMS) during solidification. These macrostructures were visualized using M.O. and analysed with LASX Office 1.4.6 software, while grain sizes were quantified utilizing ImageJ 1.8.0 software. A significant grain size reduction was observed in samples solidified with EMS, a change depicted in Table 4. Specifically, the EMS-treated samples demonstrated a marked decrease in their average grain size to less than 1 mm, notably smaller than those untreated with EMS, with grain sizes averaging 5.51 mm. An extensive collection of 600 photomicrographs, taken with an optical microscope, represented 50 images across 12 different samples, to determine the volume fraction of second-phase particles via LASX Office 1.4.6 software. These results are presented in Table 4, and the average grain size values. 

In the untreated sample A1 (Figure 3a, grain size was measured at 5.51 ± 1.91 mm. Following electromagnetic stirring (EMS) treatment at different frequencies, samples A2, A3, and A4 exhibited significantly reduced grain sizes of 0.93 ± 0.67 mm, 0.79 ± 0.54 mm, and 0.84 ± 0.57 mm, respectively, as shown in Figure 3d,g,j. This EMS treatment led to notable changes in the microstructure, with dendritic structures and a high volume of second-phase particles within interdendritic zones observed in EMS-treated samples (Figure 3c,f,i,l)). Figure 3 shows the macrostructure of samples after the casting process, solidified without and with electromagnetic stirring (EMS). The samples were chemically etched to reveal the grain. In the case of solidification without an electromagnetic field, the grains start to grow from the side walls towards the central region of the mould. The effect of electromagnetically driven melt convection on the solidified structures is shown in Figure 3d,g,j. As can be seen by comparing with Figure 3a, the growth of the grains was significantly disturbed, possibly as consequence of the redistribution of the solute concentration and dendrite fragmentation due to fluid motion. On average, the grain of the electromagnetically stirred samples is smaller than that obtained during normal solidification. Electromagnetic agitation promotes the significant refinement of Co-Cr-Mo alloy grains, casted by investment casting. In the previous work [3], it was proved that the modification of the investigation process by investing in casting, through electromagnetic stirring (EMS), during solidification allowed the decrease in the size of the Co-Cr-Mo alloy grains (innovation in the manufacture of these alloys). 

The lamellar structure visible in the untreated sample A1 (Figure 3c) contrasts sharply with the refined microstructures of the EMS-treated samples. Figure 3f illustrates the lamellar and alpha phases in sample A2 treated at 15 Hz. Sample A3, treated at 75 Hz, shows dendritic microstructures with carbides highlighted against a cobalt-rich matrix in Figure 3i. Sample A4, receive the highest frequency of 150 Hz, displayed the most significant carbide volume fraction at 8.38%, depicted in Figure 3l. SEM images provided further insight into the alloy’s structural elements. Lamellar structures were observed in Figure 3b, while Figure 3e revealed areas with interdendritic precipitates at the grain boundaries. Figure 3h,k showcased chromium-rich carbides (Cr_23_C_6_) and cobalt-rich zones, respectively, marking differences in microstructural details across the samples. A shift in carbide prevalence from M_7_C_3_ to predominantly M_23_C_6_-type carbides was noted with a reduction in carbon content to approximately 0.2%, corroborated by findings from Davis [50], Patel [11], and Zhang [51]. This transition, favouring Cr_23_C_6_ carbides due to chromium’s significant presence and affinity for carbon in the alloy, emphasizes the critical role of EMS in sculpting the microstructure through the solidification and cooling phases, offering a nuanced view of the alloy’s development.

### 3.2. Mechanical Proprieties

Samples solidified under both conditions, i.e., with the action of the EMS and without, were subjected to mechanical testing to evaluate the effect of the electromagnetic stirring on the mechanical properties of Co-Cr alloys. We also investigated the radial effects of electromagnetic fields on mechanical properties and hardness. The most common carbide in cobalt-based alloys is of the Cr_23_C_6_ type and this element, when present in large amounts, actively participates in carbide formation and is also favoured for mechanical properties as well as wear and corrosion resistance and hardness [11,50,51]. Although the Co-Cr-Mo alloy has some layered structures, its microstructure has a high carbide content, which results in higher surface hardness, as shown in Table 5.

The load–displacement curves under linear enhancement loads from 0 to 10 m.N were measured. Under the load of 10 m.N, the maximum displacement out of track for Co–Cr–Mo was 375 nm, and inside the track, it was 167 nm. The indentations were performed inside and outside of the wear tracks. Indentations were repeated in different samples to ensure good reproducibility. The hardness of samples A2, A3 and A4 had better values than samples A1 (no frequency). Further, Table 5 shows the mechanical properties calculated based on instrumented indentation data and the hardness (H), elastic modulus (E) and grain size. The best H/E value ratio was calculated as 0.035 for sample A4, although A2 (0.033) and A3 (0.033) had excellent values close to A4. Qin [52] states that hardness is considered a primary material property and defines wear resistance, and [53] the elastic modulus also influences wear behaviour. Leyland [53] mentioned that an indicator of good wear resistance is the H/E ratio; the higher the value, the better. Todorovic et al. [54] define the ratio of H to E in terms of the ‘plasticity index’ as an essential parameter for reducing wear. Hardness was the main characteristic of the material that affected its wear resistance. However, it is debatable as the H/E ratio is adequate for predicting better wear resistance [52,55] and higher strength. The H/E value of samples A2, A3 and A4 were higher than sample A1 (no frequency), in addition to the excellent hardness results, indicating that samples with a reduced grain can effectively improve the surface mechanical properties of the Co-Cr-Mo alloy, as shown in Table 5. Hardness measurements were performed after the tribocorrosion tests. Young’s modulus and the H/E ratio were calculated.

### 3.3. Tribocorrosion Behaviour

**Figure 4:**
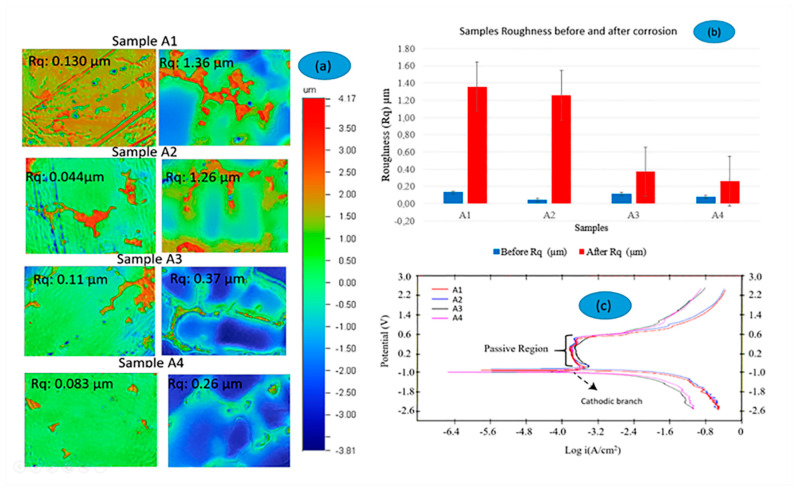
(**a**) Surface roughness morphology. (**b**) roughness comparison. (**c**) polarization curves of the Co-Cr-Mo alloy samples (A1-no EMS, A2-15 Hz, A3-75 Hz and A4-150 Hz). All samples were analysed in the Ringer lactate solution.

Figure 4 showcases three images that elucidate the tribocorrosion processes in the Co-Cr-Mo alloy samples (A1–A4). Figure 4a displays the surface roughness morphology of these samples before and after exposure to tribocorrosion in Ringer lactate solution, as captured by an optical profilometer. The images are color-coded: red represents the surface peaks, and blue signifies the valleys. Figure 4b illustrates the roughness before and after tribocorrosion tests.

Figure 4b focuses on the roughness values (Rq) of the samples before and after tribocorrosion. Here, it is notable that sample A1, which did not undergo Electro Magnetic Stimulation (EMS) treatment, exhibited the highest level of corrosion, with a roughness value of 1.36 µm. Conversely, samples A2 to A4, which benefited from EMS treatment, displayed significantly lower corrosion, underscoring the effectiveness of EMS in enhancing grain refinement and, consequently, corrosion resistance. The section also introduced the concept of passivation, which occurs either when the solubility product of the passive film is reached or due to a structural change in the film to a non-porous form. This passive behaviour is crucial for the material’s resistance, but beyond a certain point, known as the transpassive region, the protective film deteriorates.

Figure 4c, along with Table 6, compares the EMS frequency’s impact on various parameters such as corrosion current density, corrosion potential, open circuit potential (OCP), and roughness for each sample (A1-no EMS, A2-15 Hz, A3-75 Hz, and A4-150 Hz). Potentiostatic measurements depicted in this figure were performed to assess the potential’s effect on corrosion kinetics and metal ion release. Despite the differences among samples, all curves shared a similar morphology, highlighting the Co-Cr-Mo alloy’s intrinsic passive nature that favours the spontaneous formation of a protective oxide layer vital for tribocorrosion resistance.

The initial and post-tribocorrosion roughness values provide insights into the surface condition changes due to the tribocorrosion process. For instance, sample A1 showed a marked increase in roughness from 0.130 µm to 1.36 µm, indicative of significant surface degradation. In contrast, samples A2 through A4 demonstrated lesser roughness increases post-tribocorrosion, suggesting that their surfaces were better shielded, likely due to the EMS treatment’s protective influence.

This comparative analysis illustrates how the EMS treatment not only fortifies the Co-Cr-Mo alloy against corrosion but also aids in preserving surface smoothness under harsh tribocorrosion scenarios. In addition, it is important to highlight the crucial role of microstructural refinement via EMS in bolstering both the corrosion resistance and the preservation of surface integrity of biomedical implants, thereby enhancing their performance and durability.

The behaviour of the Co-Cr-Mo alloy under tribocorrosion conditions was analysed also by monitoring the open circuit potential (OCP) in both static and dynamic modes. Notably, the onset of sphere movement across the material’s surface induced a negative shift in potential across all samples, highlighting the mechanical impact on the oxide layer’s integrity. This observation aligns with Diomidis et al. [56], who noted a sudden OCP drop upon starting to slide contact, signifying potential damage to or rupture of the protective film. 

**Figure 5:**
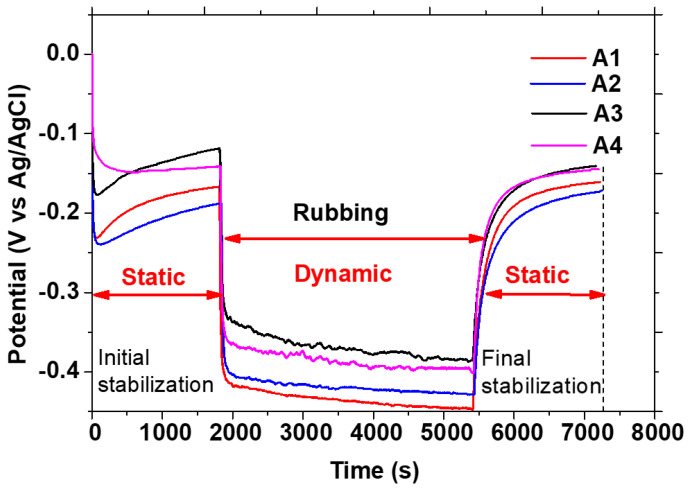
Evolution of open circuit potential (OCP vs. Ag/AgCl) values before, during, and after tribocorrosion tests, using an alumina sphere against the following samples A1, A2, A3 and A4.

Figure 5 shows the open circuit potential at air enviroment. Remarkably, sample A1 displayed significantly lower static OCP values compared to others (−0.16 mV), suggesting more pronounced passive layer degradation. The sharp OCP decline, especially evident in sample A1, indicates potential disruption of the oxide film, a phenomenon also seen when dynamic motion (rubbing) initiates.

The A2 sample (−0.19 to −0.42 V) exhibited an intermediate behaviour between A1 and A4, with its OCP partially recovering to −0.14 V, suggesting some resilience in passive layer regeneration. Sample A3 demonstrated the most robust corrosion resistance; despite an initial drop to −0.36 V during rubbing, its OCP gradually returned to −0.14 V, evidencing superior surface protection against corrosion.

These observations suggest that electromagnetic stirring (EMS), which refines grain size, plays a pivotal role in enhancing the alloy’s resistance to tribocorrosion. Sample A4, with grain sizes comparable to A3, also showed similar resilience during the rubbing phase, underscoring the importance of microstructural optimization in mitigating corrosion. Oliveira et al. [57] further corroborated these findings, indicating that a potential reduction often signals heightened corrosion susceptibility.

Comparing roughness before and after tribocorrosion, this analysis aims to elucidate the relationship between the electrochemical properties and the physical surface changes due to tribocorrosion, demonstrating how EMS treatment can mitigate surface degradation and enhance the durability of Co-Cr-Mo alloys.

To compare the corrosion current density, corrosion potential, OCP values, and roughness of the Co-Cr-Mo alloy samples, Table 6 contains the relevant data from the analysed samples. This comparison highlights how electromagnetic stirring (EMS) influences these parameters across the samples and the electrochemical characterization in static and tribocorrosion mode. The corrosion potential (Ecorr) and the corrosion current density (Icorr) were obtained from Tafel plot extrapolation [49].

Analysing the results of the corrosion potential and current density, all samples showed a significant level of corrosion activity, with sample A3 (75 Hz) and A4 (150 Hz) exhibiting slightly higher Ecorr values, indicating potentially better corrosion resistance. The Icorr values were closely grouped, suggesting that the EMS frequency had a limited effect on the corrosion current density within the tested range. 

The initial and final OCP values indicate the stability of the passive oxide layer before and after the tribocorrosion tests. Samples A2, A3, and A4 showed a recovery in OCP values post-test, implying a regeneration of the passive layer or lesser damage during testing. Sample A1 exhibited no change, suggesting potential for greater passive layer disruption.

The surface roughness: There is a clear trend that EMS treatment (A2, A3, A4) reduced the roughness increase due to corrosion compared to the untreated sample (A1). This implies that EMS-treated samples might have a more robust and adherent passive layer, contributing to their smoother surface post-corrosion. The remarkable increase in roughness for A1 post-corrosion highlights significant surface degradation, likely due to the lack of EMS treatment.

Comparing the results with EMS effectiveness, the application of EMS has evidently refined the grain size, which correlates with improved corrosion resistance and reduced roughness after corrosion. This suggests that a refined microstructure, likely with a more uniform distribution of carbides and other alloying elements, can enhance the alloy’s resistance to the tribocorrosion processes. In the others words, the use of EMS in the casting of Co-Cr-Mo alloys appears to enhance their tribocorrosion resistance, as evidenced by the improved corrosion potential, reduced corrosion current density, and minimized roughness after corrosion in EMS-treated samples. These enhancements are particularly notable in samples treated at higher frequencies (75 Hz and 150 Hz), which exhibited the most significant improvements in tribocorrosion behaviour.

**Figure 6:**
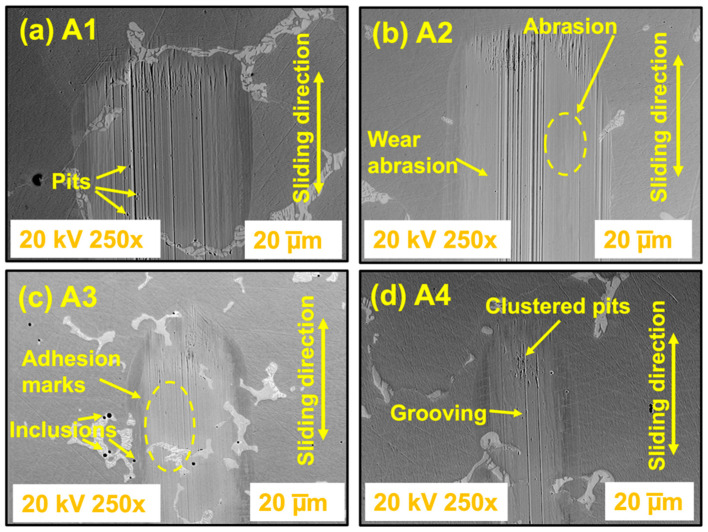
SEM images (**a**–**d**) from wear tracks. Scanning electron microscopy (SEM) images of corroded and worn surfaces in the presence of Ringer lactate solution from the samples: (**a**) A1; (**b**) A2; (**c**) A3, and (**d**) A4.

After the tribocorrosion test under the conditions of abrasive (mechanical) wear and corrosion (electrochemical), it is possible to verify the wear tracks in the samples, as shown in Figure 6. It helps us to understand the physical constitution of sample/electrolyte/sphere interactions in the wear process/corrosion. Figure 6 shows the morphology of the wear track of all samples. This alloy presented a microstructure of primary carbides dispersed in a eutectic interdendritic matrix of solid cobalt solution and many carbides and lamellar structures [58]. Figure 6 shows the wear track covered by a compacted film of oxide debris after a corrosion–wear test. Figure 6a shows the wear marks, plus many pits, and the width of the larger wear track compared to samples A3 and A4. Figure 6b exhibits increased extruded deformation lines on the wear track. Figure 6c shows adhesion marks and some inclusions from the manufacturing process. Figure 6d shows the sample with clustered pits and several grooves aligned along its length. All worn tracks show sliding grooves that can be attributed to abrasive wear. Arrows indicate the direction of sliding.

As reported in the literature [5,6,59] in the wear–corrosion system, there are two processes: (a) mechanical delamination of the passive layer in the wear track and (b) progressive electrochemical passivation of that activewear track area. We observed both processes (mechanical and electrochemical) in the samples. Some time ago, Shetty et al. [60] affirmed the existence of the relationship of carbide wear resistance, which may influence material wear not only because of their high hardness characteristics but also as a protective barrier against matrix delamination. The work of Varano et al. [61] confirmed that the results’ scatter revealed that the wear was independent of the grain size. Additionally, Rituerto et al. and Neville et al. [62,63] confirmed that the oxide layer formed by Co-Cr alloys primarily comprises chromium oxide (Cr_2_O_3_).

### 3.4. Tribocorrosion Mechanism

#### 3.4.1. Coefficient of Friction (COF) Variation under OCP Conditions

**Figure 7:**
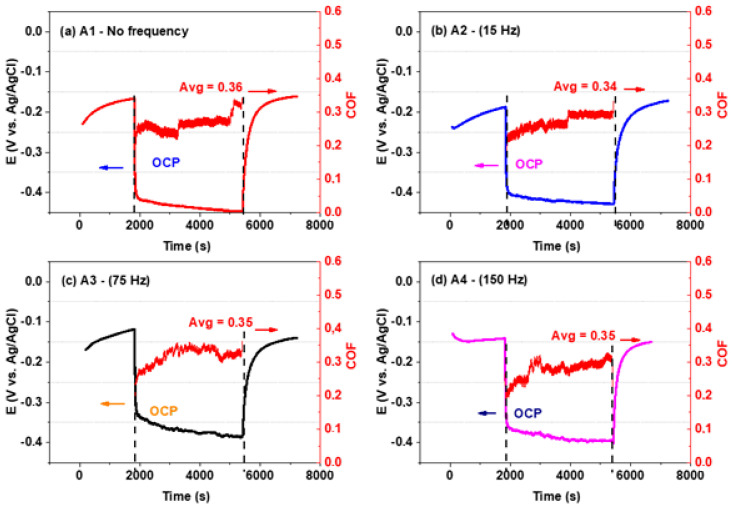
Dynamic coefficient of friction and OCP in Ringer’s lactate solution. Samples (**a**) A1 (no EMS), (**b**) A2 (15 Hz), (**c**) A3 (75 Hz) and (**d**) A4 (150 Hz).

Reciprocating sliding wear tests were conducted using the Bruker UMT Multi Specimen Test System, controlled by CETR UMT v 2.19 software, employing a ball-on-plate configuration in line with the ASTM G133 standard. The counterpart in these tests was a 4 mm diameter alumina ball with a 99% purity level, provided by Oakwade Ltd., Hartlepool, UK, to ensure an inert counterface. The friction coefficient results and the evolution of the open circuit potential (OCP vs. Ag/AgCl) during tribocorrosion tests, which involved an alumina sphere against a Co-Cr-Mo alloy in Ringer’s lactate solution, are depicted in Figure 7.

Figure 7 illustrates the variation in the friction coefficient throughout the tribocorrosion tests (from 2700 s to 5400 s), including the OCP stabilization periods before and after the wear test. An initial sharp increase in friction coefficient was observed, attributed to the surfaces’ need to adapt to each other. Notably, fluctuations in the friction coefficient were linked to debris (termed the third body) trapped between the sliding surfaces, as indicated by references [25,64]. These oscillations suggest that applying an external anodic potential may accelerate metallic surface dissolution and induce additional corrosion due to friction, a phenomenon detailed by Mischler et al. in reference [31]. This interaction also highlights the removal of a thin passive film on the surface due to mechanical wear from tribological contact.

A comparison of sample results reveals varied behaviours in the friction coefficient over time, with Figure 7a showcasing a sample without EMS, which displayed a larger grain size and a relatively high coefficient of friction (0.36 ± 0.09), indicating a significant presence of wear-induced particles. A sharp increase in the friction coefficient after 3500 s was noted, reaching an average of 0.36 ± 0.08. In contrast, Figure 7b demonstrates that the 15 Hz sample (A2) experienced a gradual increase in the friction coefficient, eventually stabilizing at 0.34 ± 0.15, despite a decrease in OCP. Similarly, the 75 Hz (A3) and 150 Hz (A4) samples, shown in Figure 7c,d, respectively, displayed a pattern of increasing friction coefficient until a certain point, after which it stabilized at 0.35 ± 0.06 and 0.35 ± 0.08, respectively. These observations are consolidated in Table 6. The OCP fluctuations noted during sliding contact for the Co-Cr-Mo alloy samples, as discussed in the literature [6,37,65], to the cyclic removal and regeneration of the passive film on the metal alloy surfaces.

Yan et al. [6] studied the corrosion-enhanced wear/tribology damage mechanisms on the wrought high-carbon Co-Cr-Mo and low-carbon Co-Cr-Mo alloys. They used three biological solutions: 50% bovine bovine serum, DMEM (Dulbecco’s modified Eagle’s medium), and NaCl solution at 37 °C. With the NaCl solution, they obtained a better result for a friction coefficient of 0.79 for the HC Co-Cr-Mo alloy. Doni et al. [65] evaluated the dry sliding and tribocorrosion behaviour of the hot-pressed Co-Cr-Mo biomedical alloy compared with the cast Co-Cr-Mo and Ti-6Al4-V alloys. The COF found was more significant than 0.40 using the NaCl electrolyte for the cast sample. Buciumeanu et al. [66] investigated the tribocorrosion behaviour of hot-pressed Co-Cr-Mo alloys in artificial saliva. They used Co-Cr-Mo square samples synthesized by a conventional hot-pressing procedure in a graphite die at three selected temperatures (900, 1000, and 1100 °C). They found the following COF (hot pressed at 900 °C = 0.37; 100 °C = 0.36 and 110 °C = 0.37). Duran et al. [67] studied the tribological characterization of a cast and SLM-processed Co-Cr-Mo alloy under wet saliva solutions. They found, for the cast alloy, a COF value of 0.48 and a wear rate of 2.27 × 10^−6^ mm^3^/N·m in saliva solution. Cassar et al. [64] reported the corrosion wear of an untreated and case-hardened Co-Cr-Mo alloy sliding against an alumina counterface in Ringer’s solution. The study shows that although the carburized alloy had a slightly higher wear loss at cathodic potential, it significantly reduced material loss when tested under both open circuit and anodic potential conditions. The COF samples were in the range of 0.65~0.55 for both specimens. Comparing this investigation, namely to tribocorrosion tests, the sample 150 Hz (A4) with reduced grain size (0.84 ± 0.57 mm) presented values of COF (0.35 ± 0.08) and rate of wear specific (k) (1.90 × 10^−6^ mm^3^/N^−1^ m^−1^) better than those presented in the literature.

#### 3.4.2. Volume of Materials Worn 

**Figure 8:**
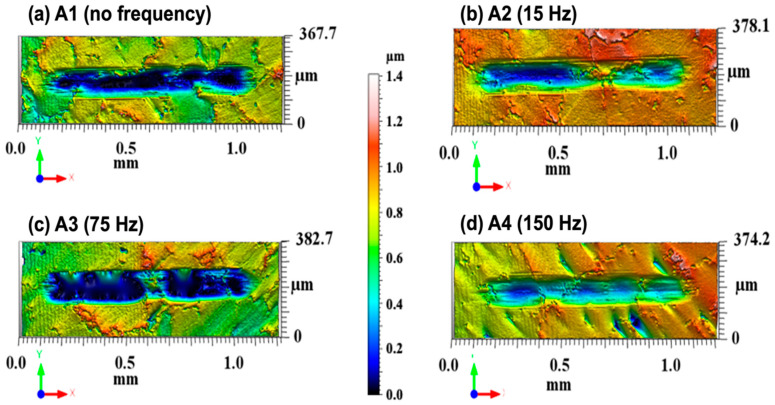
Profilometric analysis of tracks worn by tribocorrosion in Co-Cr-Mo (A1–A4) alloy samples. Wear marks were measured halfway through the track test. (**a**) is related to Sample A1; (**b**) relates to Sample A2, (**c**) relates to Sample A3, and (**d**) relates to (A4).

The applicability of the methods used to reduce materials’ degradation (wear or corrosion) could be measured by material loss. The wear profile can indicate the amount of material loss due to the area under the surface and the profile of the wear erosion [11]. The cross-sectional view can be used to determine how the wear has occurred. The alloy wear track profiles measured by the bi-dimensional profilometry of the worn samples and the corresponding wear track volume are shown in Figure 8—transversal wear profiles of the cross sections from profilometry. The track profiles presented in Figure 8 show that the tracks were A1 (~0.33 µm), A2 (~0.41 µm), A3 (~0.29 µm) and A4 (~0.44 µm) deep. It is possible to correlate the profiles of samples A1–A4 (depth × width) shown in Figure 8 with 2D interferometer images of tracks worn by tribocorrosion in samples of the Co-Cr-Mo alloy (A1–A4). Figure 8a shows that the wear tracks contain the largest wear area in the peak region, which the colour scale can identify. Figure 8b shows a different behaviour with a sharper peak. Figure 8c shows the most prominent peak with the smallest width. Figure 8d shows a shallower depth among all samples, showing a more prominent peak. A comparison of these results indicates that the 150 Hz sample presented better results in track area and worn volume than the remaining samples. 

**Figure 9:**
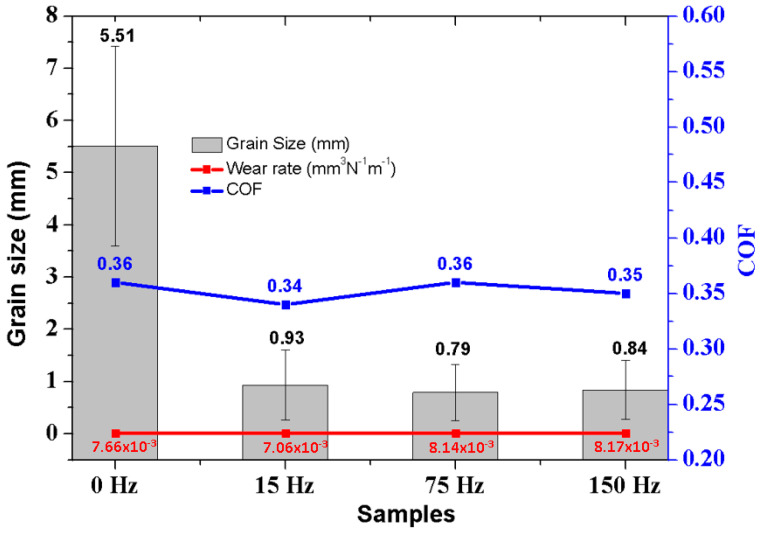
Comparative between medium grain size, wear rate and COF.

The findings regarding the coefficient of friction (COF), wear area, wear rate, and wear volume of the tracks on the Co-Cr-Mo alloy surfaces—treated with no electromagnetic stimulation (EMS) and at frequencies of 15 Hz, 75 Hz, and 150 Hz—are consolidated in Table 7. These results demonstrate that the 150 Hz sample exhibited a reduced wear profile across all examined parameters. This improvement is attributed to the enhanced wear resistance offered by the combination of the alumina sphere (Al_2_O_3_) action and the tribocorrosion process. Notably, the 150 Hz sample had the second smallest average grain size, trailing only behind the 75 Hz sample (A3). Additionally, this sample showcased a lower volume of wear (1.70 × 10^−5^ mm^3^) and a more favourable specific wear rate (1.90 × 10^−6^ mm^3^/N·m), indicating superior tribocorrosion resistance. In comparison to volumetric loss values reported in previous studies [68,69], this investigation reveals lower values. Figure 9 provides a comparative analysis of the relationships between grain size, wear rate, and COF.

Table 7 outlines the results from the tribocorrosion tests (Al_2_O_3_ sphere against Co-Cr-Mo alloy), detailing grain size, COF, specific wear rate, and worn volume.

## 4. Conclusions

This investigation into the wear properties of Co-Cr-Mo alloys processed through investment casting, both with and without the application of electromagnetic stirring (EMS), has provided substantial insights. When subjected to tribocorrosion tests in Ringer’s solution, several key findings emerged, as can be concluded hereafter.

Incorporating EMS significantly enhances the microstructural homogeneity of the alloy. It yields finer grains and increased hardness, which directly contributes to the alloy’s enhanced resistance against tribocorrosion.

Samples processed with EMS (A2, A3, and A4) exhibited superior wear rate performances due to their finer grain sizes. In particular, the application of rotating EMS post-casting significantly reduces the average grain size. This reduction, caused by the forced movement of molten metal, effectively refines grains during the solidification process and leads to a more refined microstructure and enhanced mechanical and tribocorrosion properties compared to traditional solidification methods.

Comparative analyses of the coefficient of friction (COF), wear volume, and wear rate indicate that the sample subjected to a 150 Hz EMS frequency (A4) notably outperformed those without EMS. This sample registered the lowest specific wear rate (1.90 × 10^−6^ mm^3^/N·m) and a COF of 0.35 ± 0.08.

The tribocorrosion behaviour, particularly under dynamic conditions with open circuit potential (OCP), revealed that samples with EMS-induced grain refinement (A2, A3, and A4) exhibited reduced corrosion tendencies compared to the non-EMS sample (A1). This improved wear resistance is likely a result of the optimized microstructure and the presence of secondary phases such as carbides. Additionally, the formation of a third body during friction testing could further mitigate the friction coefficient.

EMS-treated samples consistently showed superior performance in terms of COF and wear properties across the tested frequencies (75 Hz and 150 Hz). Notably, the 150 Hz treatment showcased a significant decrease in both specific wear rate and wear volume, emphasizing the effectiveness of EMS in enhancing tribocorrosion resistance.

This study highlights the profound impact of EMS on improving the wear resistance and tribocorrosion behaviour of Co-Cr-Mo alloys, underlining the technique’s potential in tailoring microstructural properties for enhanced biomedical implant performance.

## Figures and Tables

**Table 1:** Chemical composition of Co-Cr-Mo alloy (wt.%).

Plate Measurements		Chemical Composition (wt.%)								
Dimension (mm)	Thickness (mm)	Cr	Mo	Ni	Fe	C	Si	Mn	W	Co
20 × 20	2	29.9	5.53	0.3	<0.75	<0.35	<1.0	1.0	<0.2	Bal.

**Table 2:** Chemical composition of Ringer’s lactate solution.

Composition	Concentration (mg/1000 mL)
Sodium chloride (NaCl)	600
Potassium chloride (KCl)	40
Calcium Chloride dihydrate (CaCl_2_.2H_2_O)	27
Sodium lactate (NaC_3_H_5_O_3_)	312
Osmolarity (mOsm/L)	277
pH	5.0–7.5

**Table 3:** Tribocorrosion parameter test on Co-Cr-Mo alloy.

Test Parameters	Description
Alumina counterface diameter, mm	4.7
Normal load, N	5.0
Hertzian initial contact pressure, GPa	4.2
Sliding speed, mm/s	1.0
Reciprocating stroke length, mm	1.0
Frequency, Hz	1.0
Total tests duration, s/sample	7200
Sliding distance, m	3.6
Reciprocating period, s	1800
Ringer solution temperature, °C	37 ± 1
Condition	Wet, Ringer’s solution
Working electrode (WE)	Co-Cr-Mo alloy
Reference electrode (RE)	Ag/AgCl
Counter electrode (CE)	Platinum wire

**Table 4:** Different electromagnetic frequency fields (EMS), volume fraction and grain size.

EMS Frequency	Volume Fraction (%) ± (D.P.)	Grain Size (mm) ± (D.P.)
No frequency (A1)	6.86 ± 0.51	5.51 ± 1.91
15 Hz (A2)	6.53 ± 0.82	0.93 ± 0.67
75 Hz (A3)	5.50 ± 0.50	0.79 ± 0.54
150 Hz (A4)	8.38 ± 0.56	0.84 ± 0.57

**Table 5:** Mechanical properties, hardness, Young’s modulus and H/E ratio.

Sample	Hardness Outside the Wear Track (GPa)	Hardness Inside the Wear Track (GPa)	E (GPa)	H/E
A1 (No EMS)	6.28 ± 0.63	6.54 ± 0.80	213 ± 6.00	0.031
A2 (15 Hz)	6.92 ± 0.36	7.09 ± 0.51	214 ± 3.70	0.033
A3 (75 Hz)	7.00 ± 0.37	7.18 ± 0.52	218 ± 6.36	0.033
A4 (150 Hz)	7.27 ± 0.95	7.35 ± 0.88	210 ± 6.72	0.035

**Table 6:** Comparison of EMS frequency and corrosion current density, corrosion potential, OCP values, and roughness of the Co-Cr-Mo alloy samples. The colour table is just to conduct the yes and facilitate the visualization.

Sample	EMS Freq.	Corrosion				Roughness	
		Icorr (×10^−4^ A)	Ecorr (mV)	Initial OCP (mV)	Final OCP (mV)	Before Corrosion (Ra, µm)	After Corrosion (Ra, µm)
A1-No EMS	0 Hz	−3.959	−846	−0.16	−0.16	0.130	1.36
A2-15 Hz	15 Hz	−3.970	−857	−0.19	−0.14	0.04	1.26
A3-75 Hz	75 Hz	−3.880	−974	−0.11	−0.14	0.11	0.37
A4-150 Hz	150 Hz	−3.947	−985	−0.14	−0.14	0.08	0.26

**Table 7:** Grain size, COF, specific wear rate, worn volume during the tribocorrosion tests (Al_2_O_3_ sphere against Co-Cr-Mo alloy).

Sample/EMS	Grain Size (mm) SD	Mean Friction Coefficient, SD	Area of the Wear Track (mm^2^)	Specific Wear Rate (k) [mm^3^/N^−1^ m^−1^]	Volume of the Wear Track [mm^3^]
A1/No EMS	5.51 ± 1.91	0.36 ± 0.09	(4.07 ± 1 × 10^−5^)	3.30 × 10^−6^	2.97 × 10^−5^
A2 (15 Hz)	0.93 ± 0.67	0.34 ± 0.15	(3.83 ± 1 × 10^5^)	2.39 × 10^−6^	2.15 × 10^−5^
A3 (75 Hz)	0.79 ± 0.54	0.35 ± 0.06	(4.08 ± 1 × 10^−5^)	2.35 × 10^−6^	2.11 × 10^−5^
A4 (150 Hz)	0.84 ± 0.57	0.35 ± 0.08	(3.53 ± 1 × 10^−5^)	1.90 × 10^−6^	1.70 × 10^−5^

## Data Availability

Data are contained within the article.
